# FFC: a scalable FASTA compressor

**DOI:** 10.1093/bioinformatics/btag132

**Published:** 2026-03-23

**Authors:** Szymon Grabowski, Tomasz M Kowalski, Robert Susik

**Affiliations:** Institute of Applied Computer Science, Lodz University of Technology, Łódź, 90-537, Poland; Institute of Applied Computer Science, Lodz University of Technology, Łódź, 90-537, Poland; Institute of Applied Computer Science, Lodz University of Technology, Łódź, 90-537, Poland

## Abstract

**Summary:**

FASTA is a widely used text-based format for storing nucleotide and protein sequences. The existing FASTA compressors usually focus on (slightly) improving the compression ratio, not on practical performance. We present FFC, a scalable FASTA compressor that achieves average compression speeds 4.7× and 11.4× higher than two high-performance compressors, zstd and NAF, respectively, across a benchmark set of seven single genomes. It also delivers average decompression speeds 3.5× and 2.7× higher than zstd and NAF, respectively. Although a chunk-based zstd variant with parallel decompression, pzstd, almost matches FFC speed, its compression ratio is on average by 23% worse than FFC’s. For the experiment, a 14-core workstation and a RAM disk (to reduce the impact of I/O) were used.

**Availability and implementation:**

FFC is freely available at github.com/kowallus/ffc and also as a Zenodo repository at 10.5281/zenodo.18892353, and the used datasets at 10.5281/zenodo.18873744.

## 1 Introduction

Data compression techniques are popular in bioinformatics, both as a research topic and as applications (Deorowicz and Grabowski 2013, Hosseini *et al.* 2016). The rapidly growing amounts of data in genomics and related areas demand compact and practical replacements for widely used formats in order to obtain savings in storage, transmission, operating RAM memory, and sometimes also searching and computation time. Perhaps surprisingly, there is no widely accepted replacement for FASTA, a very basic format for storing the central object of computational biology, a genome. Obviously, FASTA datasets *are* stored and handled in a compressed form, but the dominating format in repositories is gzip, devised in 1992.

Let us briefly describe the FASTA format. A FASTA file contains one or more sequences, each starting with a header line, followed by DNA symbols, usually split into equal-length lines (the last line in a sequence may be shorter, though). The header usually starts with the symbol >. The header lines are variable-length, in general.

Although FASTA files are also used for storing amino acid (protein) sequences, from now on we focus on DNA data. A FASTA dataset typically contains a single genome (or a chromosome). It is well-known that DNA sequences for an individual are hardly compressible (beyond simple bit packing), and the most successful solutions (Cao *et al.* 2007, Pratas *et al.* 2019, Silva *et al.* 2020, Sousa *et al.* 2024), involving such techniques as statistical and algorithmic model mixtures, arithmetic coding, and recently also neural networks, are slow and thus impractical in common scenarios, as reported in Kryukov *et al.* (2020). According to the same benchmark, perhaps the most practical FASTA compressor, considering the compression ratio, the compression speed, and the decompression speed (taken together), is NAF (Kryukov *et al.* 2019), which is based on the general-purpose compressor zstd. NAF is basically a preprocessor, packing DNA symbols into nibbles, removing the header lines, etc., but the improvement to the baseline zstd obtained with these simple means is quite significant. On the other hand, NAF, even in its highest (and slowest) level 22, can be outperformed by ∼10% (sometimes more) by GeCo3 (Silva *et al.* 2020), yet GeCo3 needs about an order of magnitude more time for the compression. The more recent JARVIS3 (Sousa *et al.* 2024) from the same team reduces the speed gap, but still, compressing a human genome with strong modeling takes minutes rather than seconds. In this work, we present a highly optimized multithreaded tool, FFC (Fast FASTA Compressor), based on simple but DNA-dedicated ideas, attempting to obtain possibly high (de)compression speeds rather than possibly high compression ratios.

## 2 Materials and methods

**Figure 1:**
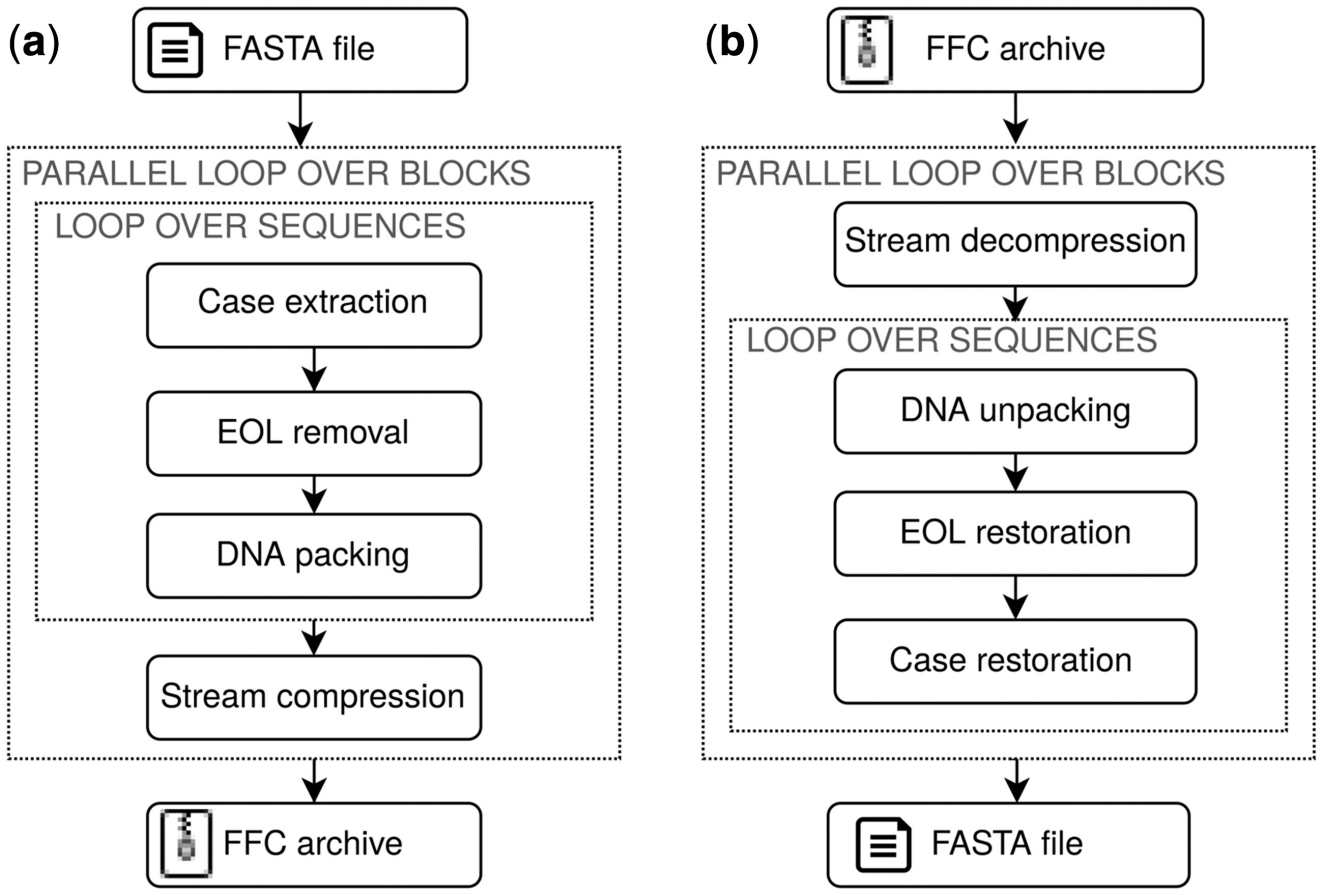
Overview of FFC pipeline. (a) Compression. (b) Decompression.

The FFC compressor processes the input file in blocks of the default size *B *= 4 MB (the last block on the input may be smaller). Each of the *t* worker threads reads the next block and processes it independently of the other ones. A high-level presentation of the successive steps of the compressor and decompressor is given in Fig. 1.

The content of each block, after uppercasing and End-Of-Line (EOL) removal (explained later), is partitioned into strings of one of four types: DNA, NNN, MIX, and RAW. For the first three of them, the string length is rounded down to a multiple of 8 for efficient bit-wise processing. DNA, NNN, and MIX are also the strings covering the sequence itself, while RAW “essentially” handles the sequence header. DNA strings are such that they contain only symbols from the standard alphabet: A, C, G, T. NNN is a string of N symbols only. MIX is a sequence of symbols that can neither be classified as DNA nor NNN; its length is arbitrary.

Assume *t* worker threads. Processing the *i*-th block, for i≥t, cannot be started before the (i−t)-th block is finished. To check if the current block starts within a sequence header, it requires reading backwards the previous block until an EOL or > symbol is found. Only in the latter case are we within a header, and then the read symbols, until the end of the header, are put in a RAW string, and the current pointer is aligned with the beginning of the sequence that follows. If, however, the block does not start within a header, the current pointer points to its first symbol. Now, the symbols from the current pointer until the end of the block or the end of the current sequence (whichever comes first) are converted to uppercase, and their case flags are preserved and packed into bytes.

In the next step, the line length, as the distance between successive EOL characters, is detected and is stored in the block’s metadata. If it remains consistent, then the EOLs are removed. This is usually the case. If not, however, the inconsistent suffix of the current sequence is kept with EOLs in the next RAW block.

After this step, parsing of the preprocessed block starts, using the string types described above. The DNA data are packed into bytes, while MIX and RAW are kept verbatim. As regards the NNN strings, only their lengths are kept. Strings of the same type are stored in separate streams, and the offsets and lengths of particular strings are kept in metadata. Finally, some of those streams may be zstd-compressed, and the compression choice for the streams is stored in metadata on the block level, as well as in the global FFC archive header. By default, the MIX and RAW streams, as well as packed case flags and block metadata are compressed with zstd-1 with enabled long-distance matching, but the DNA stream (which is usually dominating in size) is only “probed” in the first block, and only if zstd compression reduces its packed size by at least a factor 1.25 is it applied for the whole input. Due to this quick check, DNA streams from redundant datasets (e.g. virus strains) are submitted to backend compression, possibly with multi-fold reduction in the final size, while hardly redundant datasets (e.g. a human genome) are compressed at great speed.

The steps of the decompressor, which is also multithreaded, should be obvious on a high level from the given presentation of the compressor.

We put some effort into boosting the performance of the compressor and the decompressor. Testing if a given 8-byte chunk is of ACGT type is performed with a bit-wise trick involving four XOR, five AND, four add, four OR, and four negate operations, as well as one assignment and one comparison. Another bit trick is used to detect EOL symbols (0x0A) in 8-byte chunks. DNA chunks are decoded using a fast AVX2 procedure, taken from https://github.com/Daniel-Liu-c0deb0t/cute-nucleotides, by Daniel Liu (the function *bits_to_n_shuffle*), only translated from Rust to C++.

Although FFC is a FASTA-dedicated compressor, it works losslessly for any data on its input (in the case of non-DNA data, most of the input will be detected as MIX chunks and then zstd-compressed). In particular, its compression performance is not noticeably hampered on tar archives.

## 3 Results

For the experiments, we took eight compressors, including five specialized ones (Genozip, JARVIS3, MBGC, NAF, and FFC) and three general-purpose ones (igzip, zstd, and pzstd). Their versions and used command-line options are given in Data, available as supplementary data at *Bioinformatics* online. Note that Genozip (Lan *et al.* 2021) is a versatile compressor supporting all common genomic file formats but preferring high compression ratio over speed, while MBGC (Kowalski, 2026) is dedicated to collections of multiple (similar) genomes, combining high speed and high compression ratios in such scenarios. The collection of 11 FASTA datasets is quite diverse, as it comprises a few single and not very redundant large mammalian and plant genomes, as well as repetitive collections, including a large COVID-19 viral genome collection and one protein dataset. Two of those inputs (cere, hg19) are tar archives.

FFC was written in C++ and compiled with gcc 10.2.1. FFC and other compressors were tested on a Linux (Debian) machine equipped with a 14-core Intel Core i9-10940X 3.3 GHz CPU, 128 GB of DDR4-RAM (CL 16, clocked at 2666 MHz), and an SSD (ADATA 4 TB M.2 PCIe Legend 960). Each presented timing is a median over 9 runs, with cache flushing between runs. In the current section, results of two experiments are presented. Please note that we present the speeds in megabytes (MB) or gigabytes (GB) per second, but for the FFC block size, being a power of 2, it is more convenient to use, e.g. mebibytes (MiB). More results (including the impact of the block size), as well as details on the datasets and the test methodology, can be found in Data, available as supplementary data at *Bioinformatics* online.

For the test presented in Table 1, the input datasets and output archives were stored on the disk. We can see from the table that FFC is clearly the fastest tool, both in the compression and the decompression, winning in 8 out of 11 cases. Still, four other tools, all presented in the top part of the table, are also fast, with pzstd compression speed being often within 10% lower than FFC’s and even higher in one case. With regard to the decompression speed, pzstd is only a few percent slower than FFC, while zstd is about twice as slow, with the exception of highly redundant datasets, influenza, and SARS-CoV-2, where it wins by ∼10%. Of these tools, NAF usually wins over FFC in compression ratio, while igzip, zstd, and pzstd lose rather clearly. The tools from the bottom part of the table can boast higher compression ratios (which may be particularly striking for repetitive collections: cere, influenza, and SARS-CoV-2), but are also slower, in some cases (e.g. JARVIS3 in compression), by two orders of magnitude slower than FFC. Overall, we can conclude that on our test machine FFC reaches ∼2 GB/s in compression and decompression speed, and only pzstd comes close, for the price of a significantly lower compression ratio and usually more than double the CPU usage (not shown for this experiment, but see also Tables 2–5 in Data, available as supplementary data at *Bioinformatics* online, where the CPU usage patterns are similar to the runs on SSD).

**Table 1:** Compression results.

	Input size	igzip-1			zstd-1–long = 22			pzstd-1			NAF-1		
	[GB]	ratio	cspeed	dspeed	ratio	cspeed	dspeed	ratio	cspeed	dspeed	ratio	cspeed	dspeed
			[MB/s]	[MB/s]		[MB/s]	[MB/s]		[MB/s]	[MB/s]		[MB/s]	[MB/s]
canFam6	2.36	2.87	1572.7	531.3	2.94	877.0	907.3	2.94	1760.5	1999.2	4.17	303.2	966.8
cere	0.49	3.25	1411.6	531.2	3.34	823.4	882.2	3.35	1593.7	1764.5	4.42	360.6	1008.3
felCat9	2.57	2.93	1587.9	537.0	3.00	887.0	918.7	3.00	1774.1	2009.7	4.26	301.9	970.7
hg19	3.20	3.11	1592.0	565.4	3.19	909.1	943.9	3.19	1787.7	2012.6	4.56	314.0	990.7
influenza	1.43	4.90	1721.8	721.8	26.89	270.2	**2305.0**	21.43	1809.0	1957.7	32.98	414.2	1058.6
mouse	2.79	2.97	1582.7	544.0	3.07	881.5	928.5	3.06	1774.2	2003.9	4.44	306.1	977.4
panTro3	3.37	3.26	1614.7	581.8	3.35	907.2	972.5	3.35	1804.7	2020.8	4.81	315.1	992.6
SARS-CoV-2	18.83	3.92	1226.8	598.2	114.73	344.6	**2307.9**	39.50	1888.9	1994.9	308.92	359.0	1382.7
T. aestivum	4.60	3.05	1627.1	544.9	3.15	890.7	882.1	3.15	1798.7	2055.7	4.07	349.9	988.1
T. durum	3.39	3.11	1628.1	553.3	3.20	891.2	907.9	3.21	1801.3	2027.8	4.20	340.3	925.3
uniprot_sprot	0.28	2.94	1274.4	529.0	3.49	509.8	1219.0	3.42	**1475.6**	**1557.6**	4.14	292.0	700.9

		Genozip def.			JARVIS3–level 7			MBGC-m1			FFC def.		
		ratio	cspeed	dspeed	ratio	cspeed	dspeed	ratio	cspeed	dspeed	ratio	cspeed	dspeed
			[MB/s]	[MB/s]		[MB/s]	[MB/s]		[MB/s]	[MB/s]		[MB/s]	[MB/s]
canFam6	2.36	**4.37**	191.2	624.1	4.22	12.5	37.4	**4.37**	27.1	69.2	3.83	**1872.3**	**2144.6**
cere	0.49	4.89	158.9	395.2	53.19	17.2	21.3	**94.86**	475.1	641.6	4.38	**1703.6**	**1829.8**
felCat9	2.57	**4.47**	200.8	649.6	4.37	12.6	39.7	4.46	28.7	70.3	3.90	**1891.5**	**2143.7**
hg19	3.20	4.88	208.2	679.4	**4.98**	13.0	45.5	**4.98**	26.3	77.8	4.16	**1951.2**	**2191.8**
influenza	1.43	**92.94**	28.0	1050.8	41.86	18.2	45.9	53.75	220.5	371.2	29.49	**2268.4**	2133.0
mouse	2.79	4.86	197.4	661.6	**4.93**	12.9	42.5	4.76	31.9	76.4	3.95	**1921.0**	**2159.3**
panTro3	3.37	5.14	210.0	823.1	**5.23**	13.1	47.6	5.22	21.6	82.9	4.37	**1973.5**	**2177.2**
SARS-CoV-2	18.83	292.57	171.8	1810.8	274.86	18.2	68.8	**594.14**	1387.8	1360.7	116.03	**2637.5**	2085.5
T. aestivum	4.60	4.76	215.5	825.2	6.71	16.2	54.7	**7.06**	29.5	119.4	4.09	**1976.3**	**2172.1**
T. durum	3.39	4.42	222.8	844.5	**5.48**	17.1	48.3	5.47	26.9	86.4	4.12	**1968.9**	**2157.0**
uniprot_sprot	0.28	**4.70**	31.8	254.9	3.13	9.6	24.7	4.17	10.9	63.9	3.89	1401.8	**1557.6**

The columns “ratio” shows the ratio of the input to the output size. Compression/decompression speeds are denoted as “cspeed”/“dspeed”. The best results are marked bold. The FFC results are duplicated to make comparisons easier. FFC in the default mode runs with 4 threads for compression and decompression, zstd, NAF and MBGC are also multi-threaded, while the other compressors work with a single thread.

**Figure 2:**
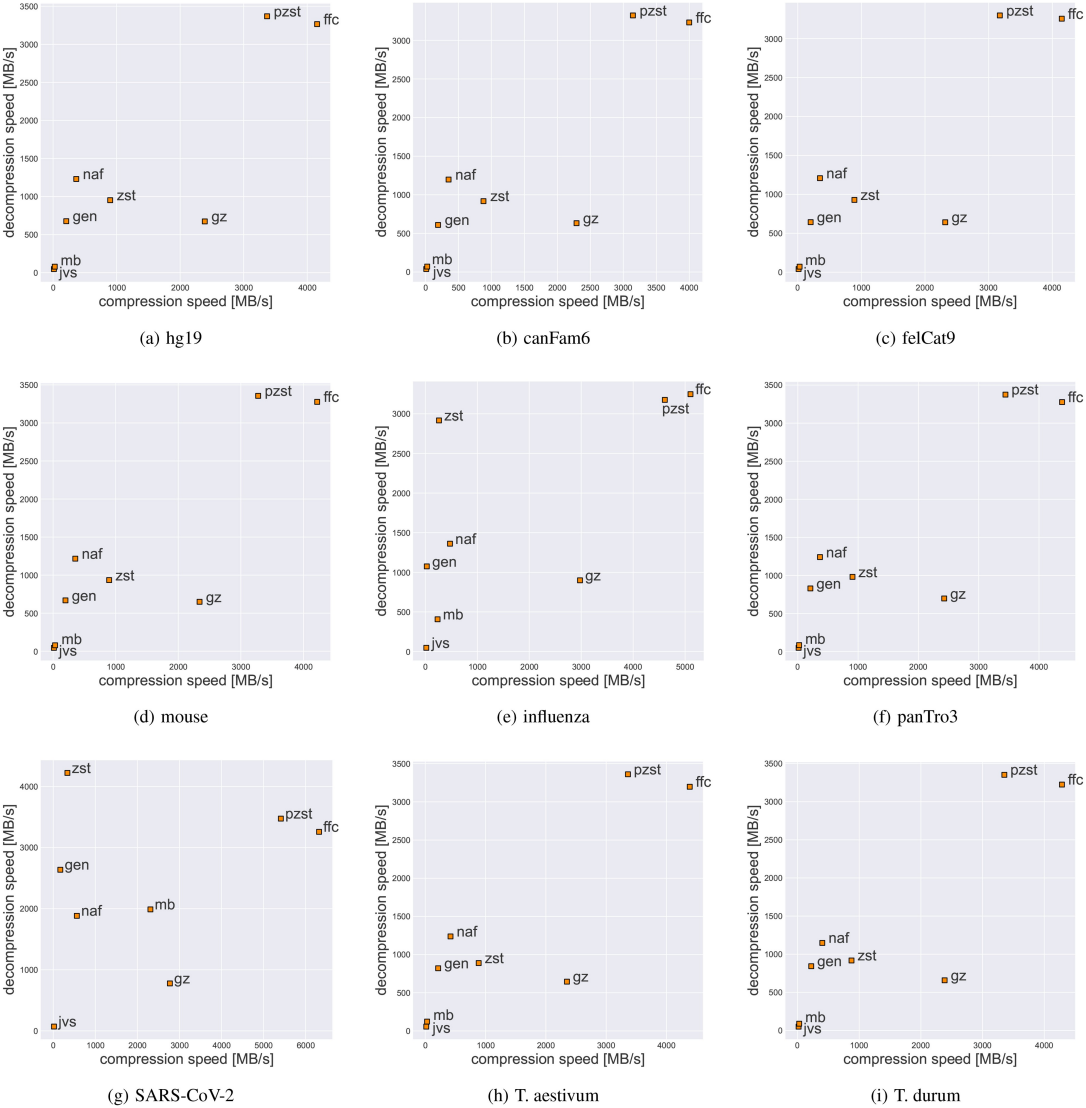
Compression speed versus decompression speed in a RAM-disk experiment. The following abbreviations are used: ffc = FFC, gen = Genozip, gz = igzip, jvs = JARVIS3, mb = MBGC, naf = NAF, pzst = pzstd, zst = zstd.

As the SSD used in our test machine is of moderate speed only (due to the PCIe 3.0 x4 interface), we decided to run a similar experiment on a RAM disk to reduce the impact of the I/O, and the results, for nine datasets, are presented in Fig. 2. Here, we focus on speed only (but the corresponding ratios can be read from Table 1). In this scenario, the advantage of FFC over the competitors, with the exception of pzstd, is even more compelling. In particular, on mammalian genomes (hg19, canFam6, mouse, panTro3), its compression (resp. decompression) speed exceeds 4 GB/s (resp. 3 GB/s).

In conclusion, we can note that FFC is comparable to pzstd in compression and decompression speed, while being significantly better in compression ratio. Also, compared to pzstd, FFC is about twice as fast on a single thread (see Table 6 in Data, available as supplementary data at *Bioinformatics* online), which translates to better energy efficiency, e.g. in a cloud setting. Compared to zstd and NAF, FFC is at least twice as fast, largely due to its multithreaded mode. In compression ratio, FFC wins easily over zstd, but loses to NAF. NAF achieves consistently higher compression ratios (partly due to the fact that it internally packs pairs rather than fours of symbols into bytes, which allows it to find more matches in a later phase). We admit that our solution in many aspects resembles NAF: symbol packing, symbol case extraction, EOL removal, and zstd backend compression, but NAF is not multithreaded and modifying it in this direction is perhaps not trivial. On the other hand, the fixed-block approach of FFC facilitates efficient multithreaded processing. FFC was designed mostly for speed, and with fast I/O, its advantage over the competitors in this aspect becomes even more pronounced; see Section 3 in Data, available as supplementary data at *Bioinformatics* online. As fast decompression can improve the overall performance of multiple bioinformatics pipelines, we hope for many successful applications of the proposed format and tool.

Future work may include introducing speed-ratio profiles, dynamic setting of the default block size with respect to the input size, adding extra backend compressors, trading zstd speed for a higher compression ratio, and a more robust heuristic in the adaptive mode. As an orthogonal change, we might extend FFC to support multiple input formats, e.g. FASTQ, creating a more versatile tool.

## Supplementary Material

btag132_Supplementary_Data

## Data Availability

The software sources, in C++, are publicly available at https://github.com/kowallus/ffc, together with FFC format specification, sample scripts and a Python FFC Decompressor compliant with FFC File Format Specification v1.0. DOI: https://doi.org/10.5281/zenodo.18892353.

## References

[btag132-B1] Cao MD , Dix TI, Allison L *et al*. A simple statistical algorithm for biological sequence compression. In: *Proceedings of the Data Compression Conference*, Snowbird, Utah, USA. p. 43–52. Los Alamitos, CA, USA: IEEE Computer Society, 2007.

[btag132-B2] Deorowicz S , Grabowski S. Data compression for sequencing data. Algorithms Mol Biol 2013;8:25. 24252160 10.1186/1748-7188-8-25 PMC3868316

[btag132-B3] Hosseini M , Pratas D, Pinho AJ. A survey on data compression methods for biological sequences. Information 2016;7:56. 10.3390/info7040056

[btag132-B4] Kowalski TM. MBGC2: boosting compression via efficient encoding of approximate matches in genome collections. GigaScience 2026;15:giag008. 41562940 10.1093/gigascience/giag008 PMC12934354

[btag132-B5] Kryukov K , Ueda MT, Nakagawa S *et al*. Nucleotide archival format (NAF) enables efficient lossless reference-free compression of DNA sequences. Bioinformatics 2019;35:3826–8. 30799504 10.1093/bioinformatics/btz144 PMC6761962

[btag132-B6] Kryukov K , Ueda MT, Nakagawa S *et al*. Sequence compression benchmark (SCB) database—a comprehensive evaluation of reference-free compressors for FASTA-formatted sequences. Gigascience 2020;9:1–12. 10.1093/gigascience/giaa072 PMC7336184 32627830

[btag132-B7] Lan D , Tobler R, Souilmi Y *et al*. Genozip: a universal extensible genomic data compressor. Bioinformatics 2021;37:2225–30. 33585897 10.1093/bioinformatics/btab102 PMC8388020

[btag132-B8] Pratas D, Hosseini M, Pinho AJ. GeCo2: an optimized tool for lossless compression and analysis of DNA sequences. In: *Proceedings of PACBB*, Ávila, Spain. p. 137–45. Cham, Switzerland: Springer International Publishing, 2019.

[btag132-B9] Silva M , Pratas D, Pinho AJ. Efficient DNA sequence compression with neural networks. Gigascience 2020;9:1–15. 10.1093/gigascience/giaa119 PMC7657843 33179040

[btag132-B10] Sousa MJP , Pinho AJ, Pratas D. JARVIS3: an efficient encoder for genomic data. Bioinformatics 2024;40:btae725. 39673739 10.1093/bioinformatics/btae725 PMC11645547

