# Supplementary material for: FFC: a scalable FASTA compressor
Source: Bioinformatics. 2026 Mar 23;42(3):btag132. doi: 10.1093/bioinformatics/btag132 (PMC13038249; doi:10.1093/bioinformatics/btag132)
Supplement: btag132_Supplementary_Data [file btag132_supplementary_data.pdf]

Supplementary Material to:
"FFC: A Scalable FASTA Compressor"

Szymon Grabowski and Tomasz M. Kowalski and Robert Susik

March 2026

1 Used datasets

Please download the following datasets and extract them (each to a separate directory). If an archive contains multiple files, they have to be concatenated to be used. Note that some of the URLs below have artificial linebreaks, so use them carefully.

1. Homo sapiens (hg19)
   `https://hgdownload.cse.ucsc.edu/goldenPath/hg19/bigZips/chromFa.tar.gz`
   (ca. 900 MB of gzipped size)

2. Mus musculus
   `https://hgdownload.cse.ucsc.edu/goldenPath/mm10/bigZips/chromFa.tar.gz`
   (ca. 830 MB of gzipped size)

3. Pan troglodytes
   `https://hgdownload.cse.ucsc.edu/goldenPath/panTro3/bigZips/panTro3.fa.gz`
   (ca. 900 MB of gzipped size)

4. Triticum aestivum
   `https://ftp.ensemblgenomes.org/pub/plants/release-22/fasta/triticum_aestivum/dna/`
   `Triticum_aestivum.IWGSP1.22.dna.genome.fa.gz`
   (ca. 1.3 GB of gzipped size)

5. Triticum durum
   `https://urgi.versailles.inra.fr/download/iwgsc/TGAC_WGS_assemblies_of_other_wheat_`
   `species/TGAC_WGS_durum_v1.fasta.gz`
   (ca. 970 MB of gzipped size)

6. Canis familiaris 6 (Dog10K_Boxer_Tasha/canFam6)
   `https://hgdownload.soe.ucsc.edu/goldenPath/canFam6/bigZips/canFam6.fa.gz`
   (725 MB of gzipped size)

7. Cat #1 Felis catus 9.0
   `https://hgdownload.soe.ucsc.edu/goldenPath/felCat9/bigZips/felCat9.fa.gz`
   (774 MB of gzipped size)

8. Saccharomyces cerevisiae (downloaded on Nov. 25, 2020)
   `https://ftp.sanger.ac.uk/pub/users/dmc/yeast/latest/cere_assemblies.tgz`
   (494 MB in FASTA)

9. Influenza (downloaded on Oct. 7, 2021)
`https://www.ncbi.nlm.nih.gov/labs/virus/vssi/#/virus?SeqType_s=Genome&VirusLineage_ss=taxid:197911&VirusLineage_ss=taxid:197912&VirusLineage_ss=taxid:197913&VirusLineage_ss=taxid:1511083`
(1,429 MB in FASTA)

10. SARS-CoV-2 (downloaded on Sep. 10, 2022)
`https://www.ncbi.nlm.nih.gov/datasets/coronavirus/genomes/`
(18,832 MB in FASTA)

11. UniProtKB Reviewed (Swiss-Prot) (downloaded on Oct. 7, 2021)
`https://ftp.uniprot.org/pub/databases/uniprot/current_release/knowledgebase/complete/uniprot_sprot.fasta.gz`
(280 MB in FASTA)

12. Homo sapiens (T2T-CHM13v2.0, complete genome)
`https://ftp.ncbi.nlm.nih.gov/genomes/all/GCF/009/914/755/GCF_009914755.1_T2T-CHM13v2.0/GCF_009914755.1_T2T-CHM13v2.0_genomic.fna.gz`
(3,156 MB in FASTA)

13. Homo sapiens (T2T-CHM13v2.0, transcript sequences)
`https://ftp.ncbi.nlm.nih.gov/genomes/all/GCF/009/914/755/GCF_009914755.1_T2T-CHM13v2.0/GCF_009914755.1_T2T-CHM13v2.0_rna.fna.gz`
(727 MB in FASTA)

14. Homo sapiens (GRCh38.p14, complete genome)
`https://ftp.ncbi.nlm.nih.gov/genomes/all/GCF/000/001/405/GCF_000001405.40_GRCh38.p14/GCF_000001405.40_GRCh38.p14_genomic.fna.gz`
(3,340 MB in FASTA)

15. Danio rerio (GRCz11, complete genome)
`https://ftp.ncbi.nlm.nih.gov/genomes/all/GCF/000/002/035/GCF_000002035.6_GRCz11/GCF_000002035.6_GRCz11_genomic.fna.gz`
(1,700 MB in FASTA)

16. Virus database (NCBI RefSeq viral genomes)
`https://ftp.ncbi.nlm.nih.gov/refseq/release/viral/viral.1.1.genomic.fna.gz`
(185 MB in FASTA)

17. Drosophila melanogaster (release 6.66, aligned genome)
`https://s3ftp.flybase.org/genomes/Drosophila_melanogaster/current/fasta/dmel-all-aligned-r6.66.fasta.gz`
(8,733 MB in FASTA)

18. Homo sapiens (GRCh38, UCSC knownCanonical exons)
`https://hgdownload.soe.ucsc.edu/goldenPath/hg38/multiz100way/alignments/knownCanonical.exonNuc.fa.gz`
(341 MB in FASTA)

Basic characteristics of the datasets are presented in Table 1. All those datasets are also available to download from `https://zenodo.org/records/18873744`.

Table 1: Datasets used in the experiments

Index	Dataset	Size (MB)	Sequences
01	*H. sapiens* (human, hg19)	3,200	93
02	*M. musculus* (mouse)	2,785	66
03	*P. troglodytes* (chimp, panTro3)	3,375	24,132
04	*T. aestivum* (common wheat)	4,605	731,921
05	*T. durum* (durum wheat)	3,386	5,671,205
06	*Canis familiaris* (dog, canFam6)	2,359	147
07	*Felis catus* (cat, felCat9)	2,572	4,508
08	*S. cerevisiae* (yeast, cere)	494	702
09	Influenza	1,429	817,587
10	SARS-CoV-2	18,832	619,750
11	UniProtKB Reviewed (Swiss-Prot)	280	565,254
12	*H. sapiens* (T2T-CHM13v2.0)	3,156	24
13	*H. sapiens* (T2T-CHM13v2.0 RNA)	727	180,091
14	*H. sapiens* (GRCh38.p14)	3,340	705
15	*D. rerio* (GRCz11)	1,700	1,922
16	Virus database (NCBI RefSeq viral)	185	20,850
17	*D. melanogaster* (release 6.66 aligned)	8,733	10,176,896
18	*H. sapiens* (GRCh38 knownCanonical exons)	341	1,470,154

2 Tested programs

igzip (version 2.31.1 from 2025-Jan-9, `https://github.com/intel/isa-l/releases/tag/v2.31.1`):

single file (e.g., FASTA or tar) compression (level -1):
`igzip -f -T28 -1 -o <archive-file> <in-file>`

decompression:
`igzip -d -T28 -o <out-file> <archive-file>`

zstd (64-bit, version 1.5.7 from 2025-Feb-19,
`https://github.com/facebook/zstd/releases/tag/v1.5.7`):

single file (e.g., FASTA or tar) compression (level -1):
`zstd -1 -T0 <in-file> -o <archive-file>`

decompression:
`zstd -d -T0 <archive-file> -o <out-file>`

Genozip (version 15.0.68 from 2024-Oct-13,
`https://github.com/divonlan/genozip/releases/tag/genozip-15.0.68`):

single FASTA file compression (default level):
`genozip <in-file> --input fasta -o <archive-file>`

decompression:
`genounzip <archive-file> -o <out-file>`

JARVIS3 (version 3.7 from 2024-Oct-28, https://github.com/cobilab/jarvis3/releases/tag/v3.7):

single FASTA file compression (default level):
```
JARVIS3.sh --input <in-file> --fasta --level 7 --threads 28
```

decompression:
```
JARVIS.sh --decompress --input <archive-file> --fasta --threads 28
```

Note that the name of the *<archive-file>* is *<in-file>* with added `tar` extension and the name of decompressed output is *<archive-file>* with added `out` extension (i.e., *<in-file>*`.tar.out`).

NAF (version 1.3.0 from 2021-May-17, https://github.com/KirillKryukov/naf):

single genomic FASTA file compression (level -1):
```
ennaf <in-file> -1 --dna -o <archive-file>
```

single protein FASTA file compression (level -1):
```
ennaf <in-file> -1 --text -o <archive-file>
```

decompression:
```
unnaf <archive-file> -o <out-file>
```

MBGC2 (version 2.1.1 from 2026-Jan-20, https://github.com/kowallus/mbgc/releases/tag/v2.1.1):

single FASTA file compression (default level, -m 1):
```
mbgc c -m1 -i <in-file> <archive-file>
```

decompression:
```
mbgc d <archive-file> <out-path>
```

FFC (version 1.1.0 from 2026-Jan-24, https://github.com/kowallus/ffc/releases/tag/v1.1, https://zenodo.org/records/18892353):

single file (e.g., FASTA or tar) compression (default level):
```
ffc -i <in-file> -o <archive-file>
```

decompression:
```
ffc -d -i <archive-file> -o <out-file>
```

pigz (version 2.8 from 2023-Aug-20,
https://github.com/madler/pigz/releases/tag/v2.8):

single file (e.g., FASTA or tar) compression (level -1):
```
pigz -f -k -1 <in-file>
```

decompression:
```
pigz -d -k <archive-file>
```

pzstd (64-bit, version 1.5.7 from 2025-Feb-19,
`https://github.com/facebook/zstd/releases/tag/v1.5.7`):

single file (e.g., FASTA or tar) compression (level -1):
`pzstd -f -1 <in-file> -o <archive-file>`

decompression:
`pzstd -d <archive-file> -o <out-file>`

Datasets *H. sapiens* (hg19) and *S. cerevisiae* (cere) are given as a collection of FASTA files. In other cases, conversion to a single file is required. The compressors igzip, zstd, and FFC accept TAR archives as input. For MBGC2, JARVIS3, Genozip and NAF, to efficiently compress datasets given as FASTA file collection, we combined them into a single Multi-Multi-FASTA file using mumu.pl (the script and more information can be found at `https://github.com/KirillKryukov/mumu`). MBGC and FFC might exhibit minor compression ratio fluctuations due to their multithreaded operation.

3 Additional results

Figs 1–4 demonstrate the impact of varying FFC parameters on its compression ratio and speed. All those experiments were run on a RAM-disk. Four multi-GB datasets were chosen: two mammalian genomes (hg19, canFam6), a large virus collection (SARS-CoV-2) and a common wheat genome (T. aestivum).

Figs 1 and 2 show how the block size affects the performance, in the default (-Ld) and -1 mode, respectively. In the default mode (level-1) zstd compression will be applied only if one of the first blocks is compressible enough. If this is the case, then the compression ratio in -Ld and -L1 should be identical (see the results on the SARS-CoV-2 dataset). If not, however, the impact from larger blocks should not always be negligible though, since a large block may be a significant part of the whole input and then even a moderate compression gain (e.g., 10%) has a slight but visible overall compression impact (cf. hg19 and canFam6). Still, the compression gain even with the largest blocks is up to about 5% (resp. 3%) on hg19 (resp. canFam6), and almost none on T. aestivum. Not surprisingly, large blocks matter a lot for highly repetitive data, which is exemplified by SARS-CoV-2; here the ratio is approximately doubled if the parameter b if changed from 22 to 30. For all four datasets, using large blocks significantly deteriorates the compression speed. Note that when the block size is, e.g., 512 MiB ($b = 29$) or 1 GiB ($b = 30$), there can be only a few threads working in parallel, which naturally hampers the overall speed. The impact of long distance matches (–long from zstd), both on compression ratio and speed, is rather tiny, with the exception of repetitive data, i.e., SARS-CoV-2.

Fig. 3 presents the compression ratios and speeds for various compression levels, directly related to the backend zstd. Note that mode 0 means that zstd compression is not applied to any data stream. On the other hand, the default/adaptive mode (Ld), which is marked on the plots with a green horizontal line, may not select zstd (-1) compression for the DNA stream, but then it uses it for the other streams. A simple conclusion is that mode 0 is never a good choice, while modes 1, 2 and the default one obtain similar speed-ratio performance. Using higher modes often implies only a minor improvement in the compression ratio and a large drop in compression speed, so it can only be recommended for repetitive collections (e.g., SARS-CoV-2), possibly together with large blocks. The usage of long distance matches has a visible (but still mild) impact in rare cases only, which is perhaps not surprising as in this experiment default block sizes, of 4 MiB, are set.

Fig. 4 focuses on the number of threads. The compression ratio is unaffected with varying the parameter t and for this reason we show only the compression and decompression speed. The compression speed improves in a practically linear manner up to $t = 8$. For a larger number of threads, some datasets (hg19, SARS-CoV-2) yet gain some improvement up to 12 or 14 threads, while the others reach a plateau.

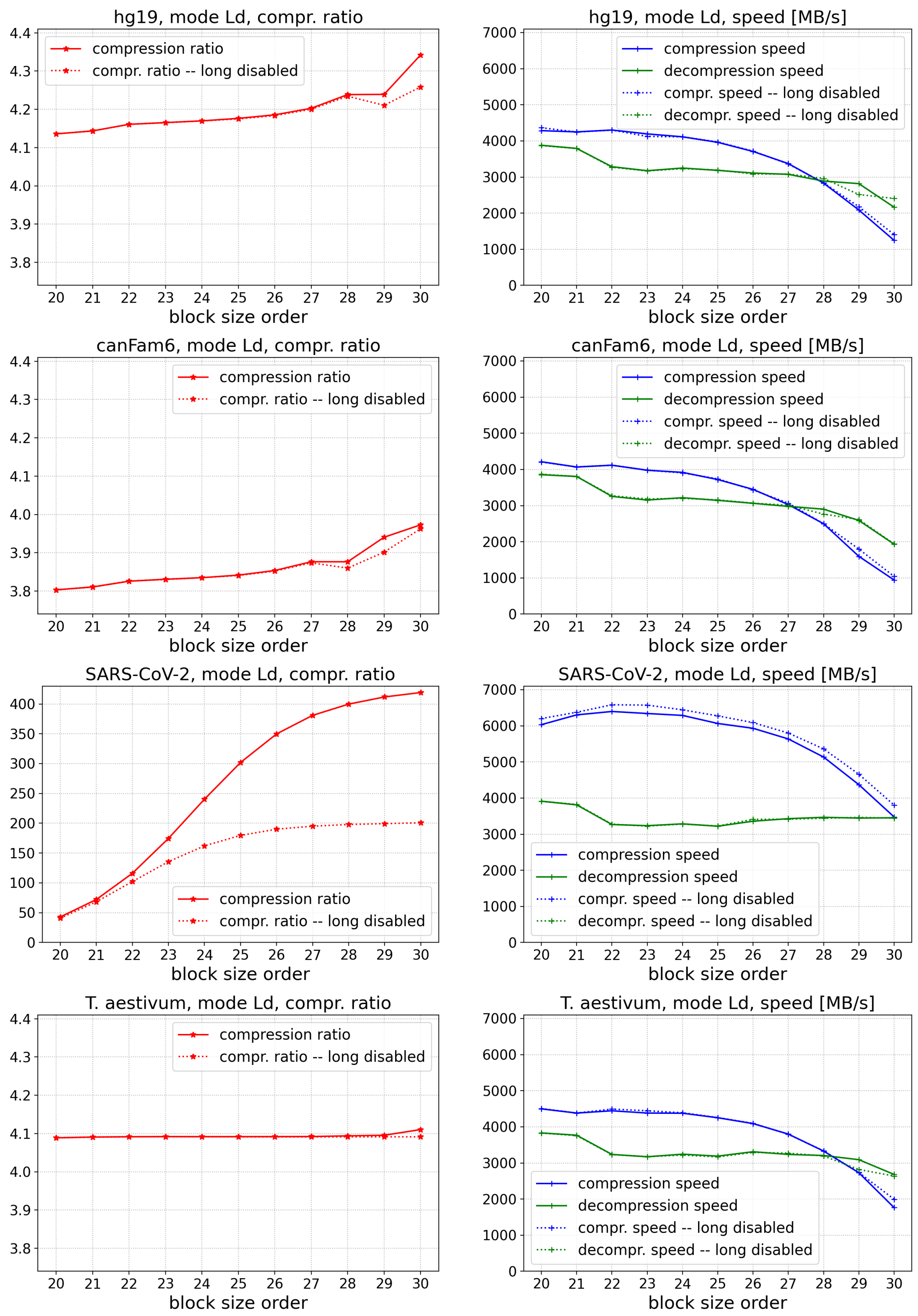

Figure 1: FFC compression ratio and compression/decompression speed with varying block size, from 2^{20} to 2^{30} bytes, in the default mode (-Ld). Tests run on a RAM-disk.

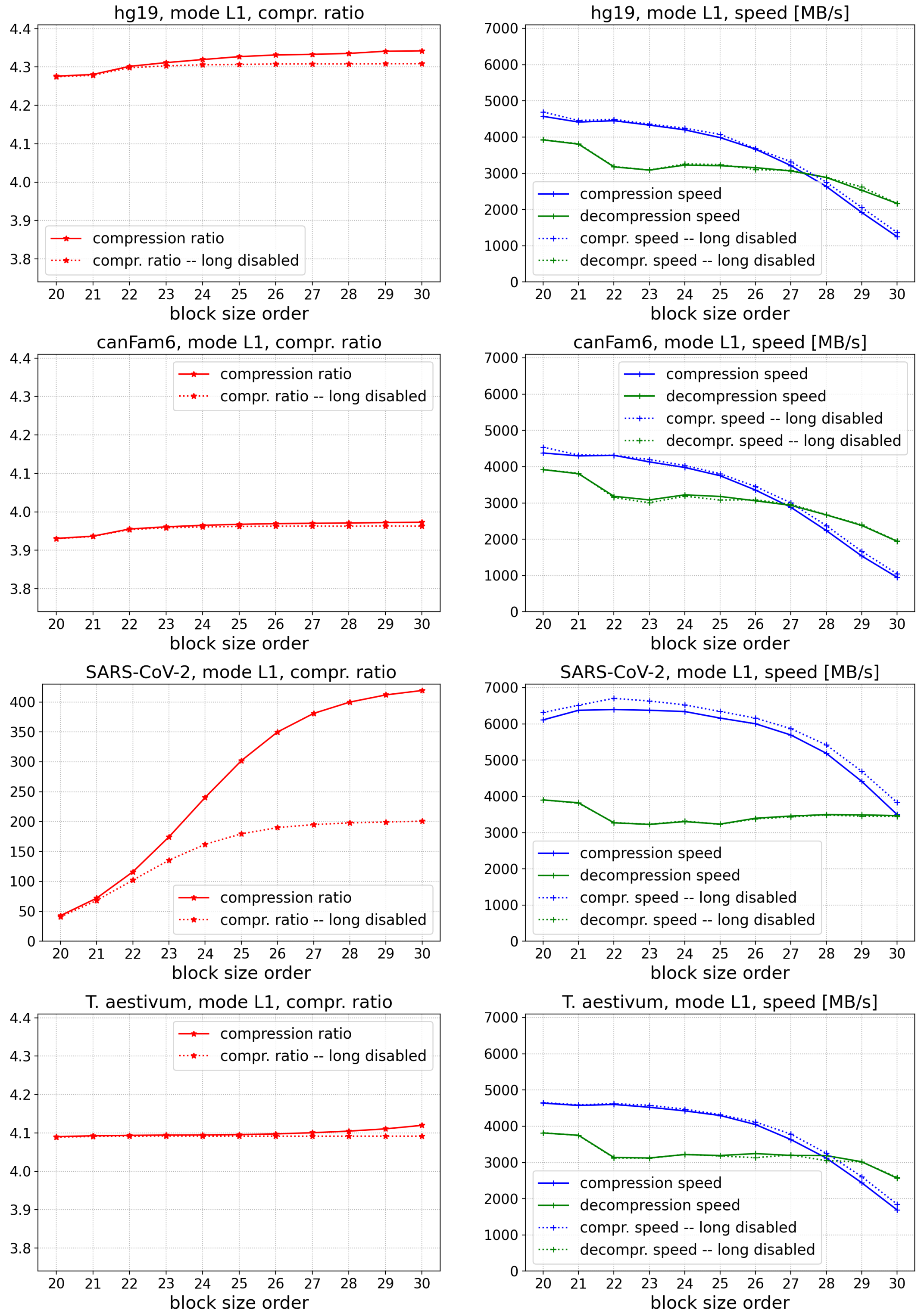

Figure 2: FFC compression ratio and compression/decompression speed with varying block size, from 2^{20} to 2^{30} bytes, in mode -L1. Tests run on a RAM-disk.

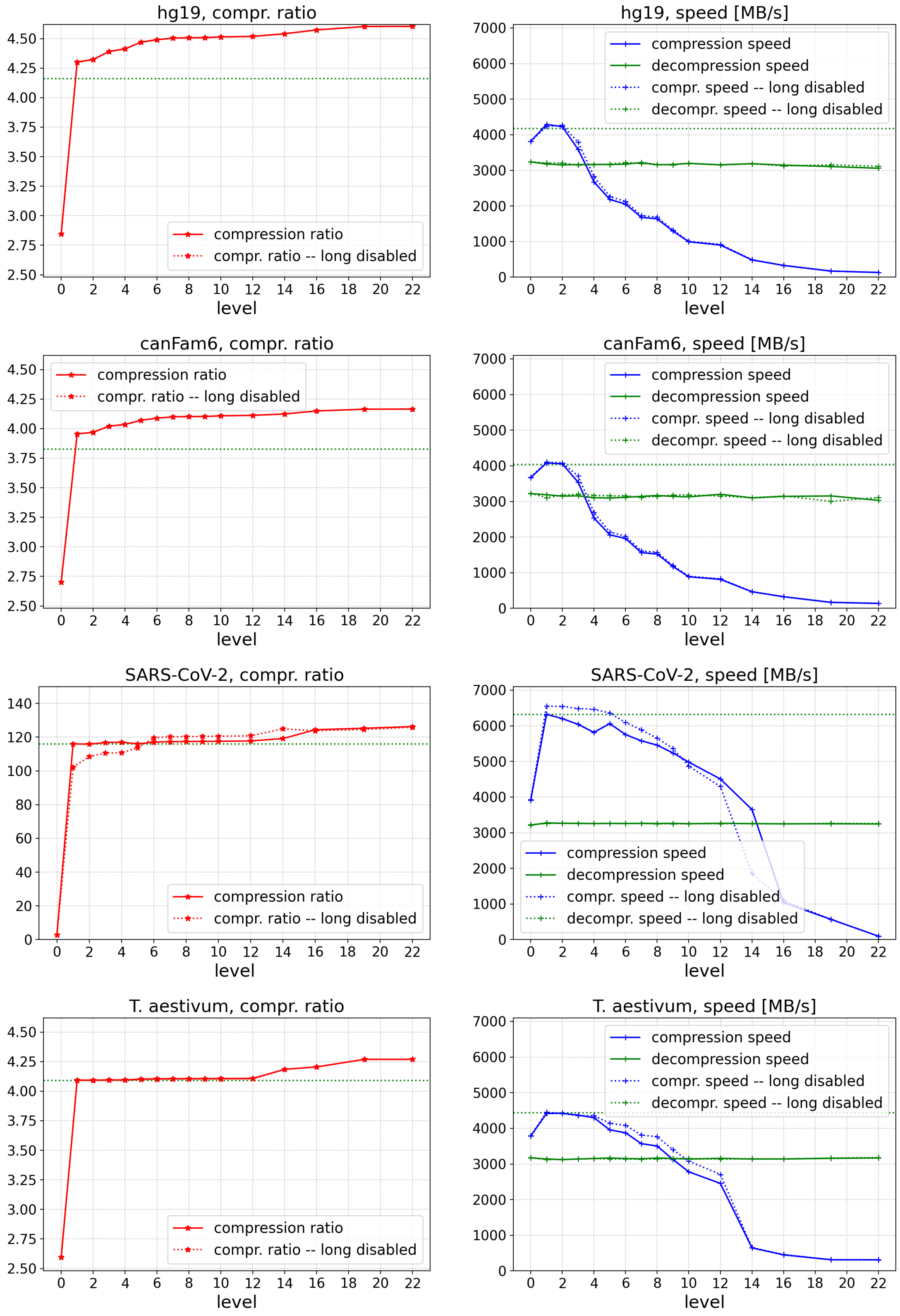

Figure 3: FFC compression ratio and compression/decompression speed with the compression level, from 0 (no zstd compression) to 22. The horizontal lines correspond to the default (Ld) mode. Tests run on a RAM-disk.

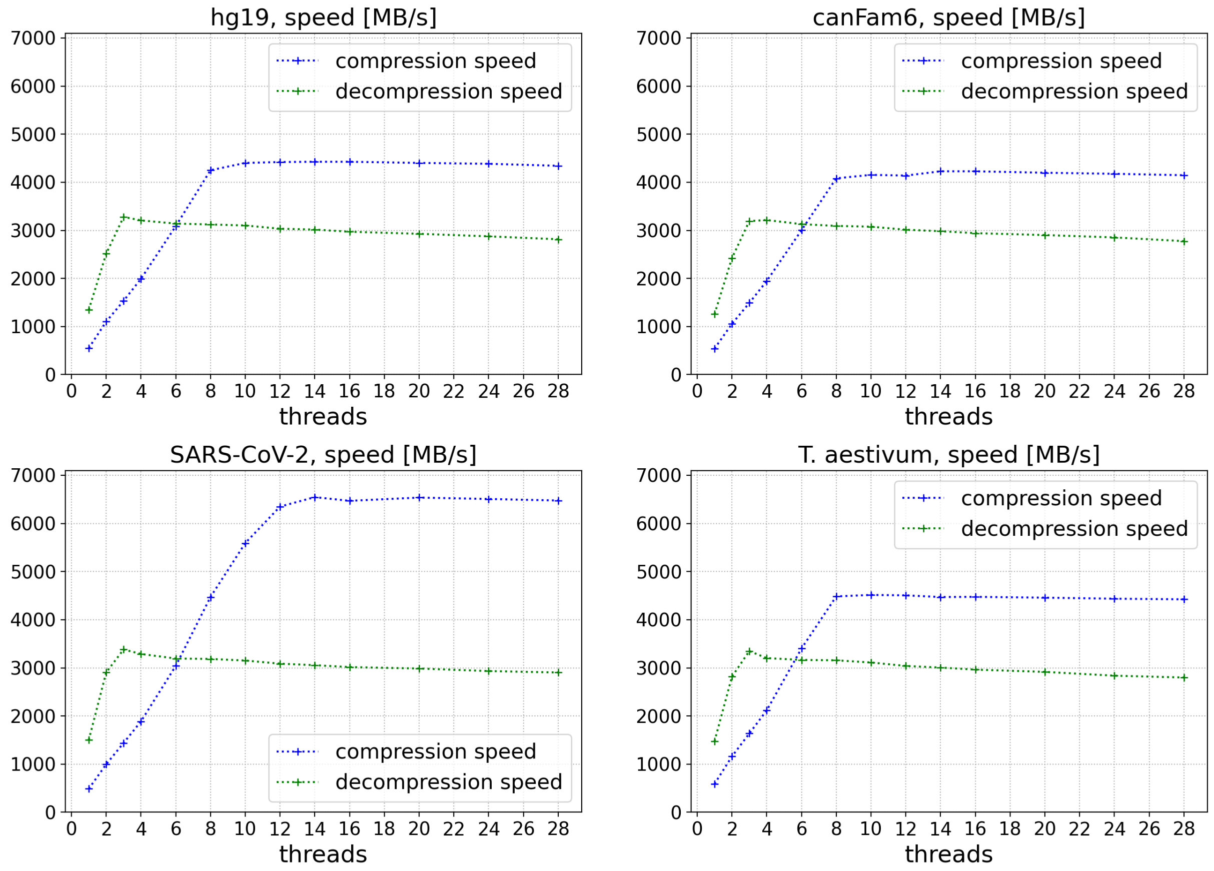

Figure 4: FFC compression/decompression speed with varying the number of threads, from 1 to 28. Tests run on a RAM-disk.

Tables 2–5 present compression ratios, speeds, as well as memory and CPU usages for the evaluated tools. A single table would be too large and for this reason we split the dataset collection (see Section 1) into four groups: mammalian, human, plant and other genomes. The experiments were performed using a RAM-disk. What distinguishes this set of experiments is not only the expanded collection of datasets, but also presenting how memory efficient the tools are, how stable compression and decompression speeds are (the standard deviations over 13 runs are given), and finally the scalability prospects of these tools, being related to the CPU usage in a multithreaded regime.

What can be seen is that most tools are generally more stable in their decompression speed (although in many cases the nominal dspeed is lower than cspeed, so this is not surprising), possibly due to lack of concurrency, e.g., in zstd. The cspeed and dspeed standard deviations are highest for FFC, often followed by pzstd, which again is not very surprising, as those are the fastest tools. Memory usages in almost all cases were below 1 GB, with a notable exception of MBGC, which can spend up to about 26 GB in compression and 10 GB in decompression. CPU usage patterns are quite interesting; pigz and pzstd use well over 2000% CPU time in compression, on our 14c/28t machine, which contrasts with about 600%–700% for FFC. This suggests that the RAM-disk scenario is still limiting for FFC, and on an SSD with very fast I/O (or using a tentative more efficient RAM-disk software) it could boost its speed even more. Decompression is even less scalable, with often about 500% dcpu for pzstd and twice less for FFC.

As there is a long-term trend of fast and relatively inexpensive storage on the market, in the experiment presented in Table 6 we wanted to reduce the impact of I/O operations even more (note also that RAM-disk devices do not achieve raw RAM speed in practice, due to various software and OS-related overheads). To this end, we read the already cached input from the SSD, yet without flushing the disk cache, and also direct the output to /dev/null. This setup should particularly strongly accentuate the differences in CPU times between competing tools.

We compared only the two fastest tools: pzstd and FFC, using 1, 4, 8, 12 and 28 threads, both in compression and decompression tests. In compression speed, FFC is often (mammalian and plant genomes) about twice faster on a single thread, and with slightly less edge (e.g., factor 1.7 or 1.8) using 28 threads. On highly repetitive datasets, influenza and SARS-CoV-2, pzstd is 2–4 times faster with a single thread, but reaches similar or worse speed with 28 threads. In decompression, in some cases FFC is about twice faster than pzstd no matter the number of threads used (perhaps with the most striking examples with 4 threads—over 6 times faster on the mouse dataset). On the overall, with the exception of 3 relatively small datasets (knownCanonical.exonNuc, uniprot_sprot, VDB), FFC exceeds 12 GB/s (and often 20 GB/s) decompression speed with 28 threads. There a few cases where pzstd wins though: SARS-CoV-2, influenza, D. melanogaster, knownCanonical.exonNuc, uniprot_sprot). Note also that on SARS-CoV-2 and influenza we measured higher FFC decompression speeds with few threads (8 and 12, respectively) and with those choices FFC takes the lead.

Although it could be said that even in this scenario pzstd is quite often not that far from FFC in compression or decompression speed, it usually loses significantly in compression ratio, see the column "ratio rel. zstd" in the top part of the table. Taking all these into account, we believe that FFC is a much better overall choice for genome storage and handling.

Table 2: Compression results on mammalian genomes, experiments on a RAM-disk

	ratio	cspeed [MB/s]	cspeed std.dev.	dspeed [MB/s]	dspeed std.dev.	cmem [GB]	dmem [GB]	ccpu [%]	dcpu [%]
canFam6 (2.36 GB)									
pigz -1	2.73	1340	3.8	326	2.3	0.03	0.00	2741	135
igzip -1	2.87	2268	38.6	631	2.0	0.05	0.06	588	100
zstd -1	2.94	2564	43.1	897	5.4	0.06	0.00	1272	128
zstd -1 –long=22	2.94	880	13.1	918	2.2	0.06	0.01	510	129
pzstd -1	2.94	3145	50.9	3323	39.6	0.14	0.14	2458	541
NAF –dna -1	4.17	351	6.2	1191	11.6	0.01	0.02	99	99
NAF –dna -1 –long 22	4.18	259	0.2	1222	4.8	0.01	0.02	99	99
JARVIS def.	4.22	13	0.0	38	0.2	0.34	0.34	295	673
MBGC def.	4.37	27	0.2	71	0.1	19.64	7.72	322	195
FFC def.	3.83	3998	178.6	3232	30.6	0.11	0.04	689	226
FFC –disable-long	3.83	4067	34.8	3232	21.9	0.10	0.04	694	225
felCat9 (2.57 GB)									
pigz -1	2.78	1361	4.0	331	2.0	0.03	0.00	2739	135
igzip -1	2.93	2276	37.8	641	1.7	0.05	0.06	582	100
zstd -1	3.00	2572	38.1	909	1.2	0.06	0.01	1265	128
zstd -1 –long=22	3.00	890	8.8	929	1.9	0.06	0.01	503	129
pzstd -1	3.00	3216	34.7	3298	32.9	0.14	0.14	2455	540
NAF –dna -1	4.26	350	2.5	1208	9.5	0.01	0.03	99	99
NAF –dna -1 –long 22	4.26	258	0.6	1225	6.0	0.01	0.03	99	99
JARVIS def.	4.37	13	0.0	41	0.2	0.34	0.34	293	701
MBGC def.	4.46	29	0.2	72	0.1	21.67	8.33	206	195
FFC def.	3.90	4149	162.1	3256	22.8	0.11	0.04	698	224
FFC –disable-long	3.90	4149	55.5	3216	35.8	0.11	0.04	698	224
mouse (2.79 GB)									
pigz -1	2.82	1372	4.3	332	1.7	0.03	0.00	2732	135
igzip -1	2.97	2302	38.1	649	4.5	0.05	0.06	579	100
zstd -1	3.06	2579	29.0	913	3.0	0.06	0.01	1262	128
zstd -1 –long=22	3.07	896	10.3	935	2.2	0.06	0.01	499	129
pzstd -1	3.06	3277	36.4	3356	57.5	0.14	0.13	2493	534
NAF –dna -1	4.44	355	1.7	1216	7.8	0.01	0.03	99	99
NAF –dna -1 –long 22	4.55	262	0.3	1238	3.9	0.01	0.03	99	99
JARVIS def.	4.93	13	0.0	44	1.3	0.34	0.34	287	713
MBGC def.	4.76	32	0.3	78	0.0	22.62	8.74	279	195
FFC def.	3.95	4220	160.9	3277	14.3	0.11	0.04	709	223
FFC –disable-long	3.95	4220	40.6	3277	23.0	0.10	0.04	710	222
panTro3 (3.37 GB)									
pigz -1	3.09	1467	6.3	357	1.7	0.03	0.00	2716	136
igzip -1	3.26	2411	32.8	697	1.3	0.05	0.06	562	100
zstd -1	3.35	2722	30.9	961	1.0	0.06	0.01	1221	129
zstd -1 –long=22	3.35	900	9.8	981	2.1	0.06	0.01	472	130
pzstd -1	3.35	3479	38.7	3375	25.1	0.14	0.13	2398	517
NAF –dna -1	4.81	368	0.7	1241	5.7	0.01	0.03	99	99
NAF –dna -1 –long 22	4.82	269	3.2	1250	5.1	0.02	0.03	99	99
JARVIS def.	5.23	13	0.0	49	0.2	0.34	0.34	276	750
MBGC def.	5.22	22	0.1	84	0.1	26.29	10.13	155	196
FFC def.	4.37	4383	176.5	3276	20.4	0.12	0.05	710	222
FFC –disable-long	4.37	4383	38.6	3276	20.4	0.12	0.05	710	222
Danio rerio, zebrafish (1.7 GB)									
pigz -1	2.72	1339	3.9	321	2.5	0.03	0.00	2735	134
igzip -1	2.86	2267	19.5	632	2.3	0.05	0.06	589	100
zstd -1	3.00	2576	46.4	929	4.4	0.06	0.00	1255	130
zstd -1 –long=22	3.00	863	18.0	945	1.5	0.06	0.01	486	131
pzstd -1	3.00	3208	43.7	3334	37.8	0.14	0.12	2427	537
NAF –dna -1	4.14	337	6.2	1076	8.0	0.01	0.04	99	99
NAF –dna -1 –long 22	4.16	247	2.8	1076	7.4	0.01	0.04	99	99
JARVIS def.	4.93	29	0.1	33	0.3	0.34	0.34	499	525
MBGC def.	5.54	38	1.0	87	0.2	12.62	4.73	336	200
FFC def.	3.50	4147	190.0	3270	34.6	0.11	0.05	758	239
FFC –disable-long	3.49	4147	47.4	3270	23.2	0.11	0.04	754	238

The column "ratio" shows the ratio of the input to the output size. Median compression / decompression speeds are denoted as "cspeed" / "dspeed", and are followed by their standard deviations over 13 runs. Compression and decompression peak memory usage is reported in columns "cmem" and "dmem", respectively, while CPU usage during compression and decompression in columns "ccpu" and "dcpu".

Table 3: Compression results on human genomes, experiments on a RAM-disk

	ratio	cspeed [MB/s]	cspeed std.dev.	dspeed [MB/s]	dspeed std.dev.	cmem [GB]	dmem [GB]	ccpu [%]	dcpu [%]
CHM13v2.0_genomic (3.16 GB)									
pigz -1	2.77	1355	2.9	327	1.9	0.03	0.00	2745	134
igzip -1	2.91	2223	19.1	645	1.6	0.05	0.06	577	100
zstd -1	3.15	2630	24.1	926	2.5	0.06	0.00	1251	130
zstd -1 –long=22	3.16	801	16.6	951	1.4	0.06	0.01	455	130
pzstd -1	3.15	3322	37.4	3358	20.6	0.14	0.13	2452	528
NAF –dna -1	4.33	354	6.8	1115	4.2	0.01	0.07	99	99
NAF –dna -1 –long 22	4.35	258	2.3	1115	5.0	0.01	0.07	99	99
JARVIS def.	4.63	28	0.1	47	0.4	0.34	0.34	523	799
MBGC def.	4.58	30	0.2	73	0.1	25.27	9.98	315	198
FFC def.	3.56	3995	145.1	3254	23.1	0.11	0.04	726	238
FFC –disable-long	3.56	3995	43.6	3254	33.6	0.11	0.04	725	237
CHM13v2.0_rna (0.73 GB)									
pigz -1	3.14	1426	10.7	351	2.7	0.03	0.00	2696	137
igzip -1	3.54	2273	33.1	686	2.5	0.05	0.06	520	100
zstd -1	5.90	3828	168.6	1347	6.8	0.06	0.00	1077	140
zstd -1 –long=22	5.94	582	26.8	1372	12.2	0.06	0.01	262	141
pzstd -1	5.88	3828	0.0	3030	63.1	0.11	0.13	1330	412
NAF –dna -1	8.06	460	12.3	1399	13.7	0.01	0.03	99	99
NAF –dna -1 –long 22	8.29	322	4.0	1372	17.0	0.02	0.03	100	99
JARVIS def.	13.38	20	0.1	24	0.1	0.34	0.34	299	260
MBGC def.	12.87	96	0.4	198	1.1	3.25	1.25	237	217
FFC def.	4.10	3828	153.5	3162	36.5	0.13	0.04	588	198
FFC –disable-long	4.09	3828	71.9	3162	63.3	0.12	0.05	594	199
hg19 (3.2 GB)									
pigz -1	2.95	1422	5.4	345	1.8	0.03	0.00	2723	135
igzip -1	3.11	2353	26.4	674	2.7	0.05	0.06	570	100
zstd -1	3.19	2645	26.8	933	3.5	0.06	0.01	1237	129
zstd -1 –long=22	3.19	899	14.7	952	2.3	0.06	0.01	488	129
pzstd -1	3.19	3368	37.5	3368	37.6	0.14	0.14	2435	524
NAF –dna -1	4.56	368	2.8	1231	4.9	0.01	0.03	99	99
NAF –dna -1 –long 22	4.59	268	0.2	1240	5.4	0.01	0.03	99	99
JARVIS def.	4.98	13	0.0	47	0.2	0.34	0.34	285	756
MBGC def.	4.97	27	0.9	79	0.1	23.38	9.47	267	197
FFC def.	4.16	4211	185.5	3265	34.6	0.11	0.04	689	221
FFC –disable-long	4.16	4267	36.8	3265	19.9	0.11	0.04	697	221
GRCh38.p14_genomic (3.34 GB)									
pigz -1	2.85	1380	2.5	335	1.6	0.03	0.00	2738	135
igzip -1	3.00	2272	19.3	659	1.6	0.05	0.06	574	100
zstd -1	3.19	2630	26.7	936	2.0	0.06	0.01	1236	130
zstd -1 –long=22	3.20	865	13.8	957	2.2	0.06	0.01	476	130
pzstd -1	3.19	3340	32.8	3340	22.1	0.14	0.13	2427	524
NAF –dna -1	4.37	357	8.3	1128	5.0	0.01	0.07	99	99
NAF –dna -1 –long 22	4.40	260	4.0	1132	5.4	0.01	0.07	99	99
JARVIS def.	4.75	26	0.1	49	0.3	0.34	0.34	479	803
MBGC def.	4.94	32	0.2	78	0.1	25.49	10.02	267	196
FFC def.	3.75	4123	147.8	3242	22.3	0.11	0.04	720	235
FFC –disable-long	3.75	4123	47.1	3274	20.6	0.10	0.04	721	234
knownCanonical.exonNuc (0.34 GB)									
pigz -1	3.81	1703	0.0	421	2.9	0.03	0.00	2601	144
igzip -1	4.11	2271	39.4	774	6.8	0.05	0.06	469	100
zstd -1	5.02	3407	135.8	1032	0.0	0.05	0.00	1088	132
zstd -1 –long=22	5.15	643	47.1	1065	9.5	0.05	0.01	304	133
pzstd -1	5.02	3407	116.3	3097	124.0	0.09	0.11	1422	494
JARVIS def.	7.79	14	0.2	14	0.0	0.33	0.33	151	114
FFC def.	5.21	2433	70.3	2271	134.7	0.18	0.07	892	296
FFC –disable-long	5.18	2433	94.8	2271	111.8	0.18	0.06	877	312

The column "ratio" shows the ratio of the input to the output size. Median compression / decompression speeds are denoted as "cspeed" / "dspeed", and are followed by their standard deviations over 13 runs. Compression and decompression peak memory usage is reported in columns "cmem" and "dmem", respectively, while CPU usage during compression and decompression in columns "ccpu" and "dcpu".

Table 4: Compression results on plant genomes, experiments on a RAM-disk

	ratio	cspeed [MB/s]	cspeed std.dev.	dspeed [MB/s]	dspeed std.dev.	cmem [GB]	dmem [GB]	ccpu [%]	dcpu [%]
cere (0.49 GB)									
pigz -1	3.08	1412	0.0	348	3.0	0.03	0.00	2677	137
igzip -1	3.25	2148	54.1	650	5.2	0.05	0.06	534	100
zstd -1	3.35	2600	62.5	898	6.0	0.06	0.00	1189	128
zstd -1 –long=22	3.34	852	33.6	915	6.9	0.06	0.01	458	129
pzstd -1	3.35	2906	70.8	3088	79.7	0.13	0.12	1999	536
NAF –dna -1	4.42	433	8.1	1335	16.9	0.01	0.01	99	99
NAF –dna -1 –long 22	4.44	311	1.3	1335	21.9	0.01	0.01	99	99
JARVIS def.	53.19	17	0.4	22	0.0	0.34	0.34	216	178
MBGC def.	93.63	555	14.7	797	6.8	1.28	0.37	502	222
FFC def.	4.38	3800	169.2	3088	79.2	0.10	0.04	565	192
FFC –disable-long	4.38	3529	140.9	3088	57.1	0.10	0.04	557	194
T. aestivum (4.6 GB)									
pigz -1	2.90	1383	2.1	328	2.0	0.03	0.00	2757	135
igzip -1	3.05	2337	26.6	645	0.7	0.05	0.06	595	100
zstd -1	3.15	2616	41.5	870	19.7	0.06	0.00	1283	127
zstd -1 –long=22	3.15	894	11.1	889	1.1	0.06	0.01	503	127
pzstd -1	3.15	3361	25.7	3361	44.1	0.14	0.13	2571	553
NAF –dna -1	4.07	419	14.2	1241	12.9	0.02	0.10	99	99
NAF –dna -1 –long 22	4.08	301	4.5	1265	5.7	0.02	0.11	99	99
JARVIS def.	6.71	16	0.1	57	0.6	0.34	0.34	368	978
MBGC def.	7.06	30	0.0	123	0.2	25.27	6.21	288	242
FFC def.	4.09	4385	233.5	3198	26.3	0.14	0.05	646	199
FFC –disable-long	4.09	4385	63.8	3220	20.3	0.13	0.05	646	199
T. durum (3.39 GB)									
pigz -1	2.96	1411	3.5	336	2.1	0.03	0.00	2750	135
igzip -1	3.11	2368	27.3	658	1.1	0.05	0.06	588	100
zstd -1	3.20	2667	45.8	898	24.0	0.06	0.00	1279	128
zstd -1 –long=22	3.20	880	12.5	918	1.9	0.06	0.01	491	128
pzstd -1	3.21	3353	31.5	3353	26.0	0.14	0.13	2512	540
NAF –dna -1	4.20	403	15.1	1148	10.1	0.02	0.32	99	99
NAF –dna -1 –long 22	4.20	294	0.4	1172	4.2	0.02	0.32	99	99
JARVIS def.	5.48	17	0.2	50	0.2	0.34	0.34	359	786
MBGC def.	5.47	27	0.1	88	0.2	19.29	4.68	265	217
FFC def.	4.12	4287	160.6	3225	25.1	0.16	0.06	754	241
FFC –disable-long	4.12	4287	34.4	3225	51.9	0.16	0.06	759	241

The column “ratio” shows the ratio of the input to the output size. Median compression / decompression speeds are denoted as “cspeed” / “dspeed”, and are followed by their standard deviations over 13 runs. Compression and decompression peak memory usage is reported in columns “cmem” and “dmem”, respectively, while CPU usage during compression and decompression in columns “ccpu” and “dcpu”.

Table 5: Compression results on other genomes, experiments on a RAM-disk

	ratio	cspeed [MB/s]	cspeed std.dev.	dspeed [MB/s]	dspeed std.dev.	cmem [GB]	dmem [GB]	ccpu [%]	dcpu [%]
D. melanogaster (8.73 GB)									
pigz -1	3.45	1676	2.1	399	1.4	0.03	0.00	2745	141
igzip -1	3.79	2746	14.6	809	2.3	0.05	0.06	537	100
zstd -1	4.96	4139	36.3	1382	3.0	0.06	0.01	1209	141
zstd -1 –long=22	5.74	446	6.4	1579	1.6	0.06	0.01	217	146
pzstd -1	4.93	5019	36.5	3398	21.0	0.13	0.13	2096	394
JARVIS def.	12.60	12	0.1	56	0.7	0.34	0.34	225	556
FFC def.	7.04	4962	181.0	3153	18.5	0.16	0.06	1087	265
FFC –disable-long	6.84	5048	40.7	3164	27.8	0.16	0.06	1016	264
influenza (1.43 GB)									
pigz -1	3.69	1662	5.3	411	2.5	0.03	0.00	2703	143
igzip -1	4.90	2977	30.5	899	3.6	0.05	0.06	502	100
zstd -1	22.44	5716	318.4	2422	25.2	0.05	0.01	663	163
zstd -1 –long=22	26.89	262	4.6	2917	28.0	0.05	0.01	131	173
pzstd -1	21.43	4610	290.4	3176	67.3	0.04	0.14	597	289
NAF –dna -1	32.98	473	3.0	1323	30.2	0.02	0.13	99	99
NAF –dna -1 –long 22	41.07	316	0.4	1429	8.0	0.02	0.14	99	100
JARVIS def.	41.86	18	0.1	47	0.7	0.34	0.33	255	405
MBGC def.	53.68	235	1.7	407	1.2	2.99	1.08	393	218
FFC def.	29.49	5104	228.8	3248	42.6	0.13	0.05	1046	222
FFC –disable-long	28.50	5293	95.7	3248	42.6	0.12	0.05	873	227
SARS-CoV-2 (18.83 GB)									
pigz -1	2.96	1405	1.6	335	1.5	0.03	0.00	2759	135
igzip -1	3.92	2769	7.5	777	1.7	0.05	0.06	556	100
zstd -1	49.14	5830	260.7	3722	22.9	0.04	0.01	452	187
zstd -1 –long=22	114.73	336	3.3	4222	34.3	0.07	0.01	121	193
pzstd -1	39.50	5412	271.8	3475	13.4	0.04	0.16	437	218
NAF –dna -1	308.92	562	1.7	1868	18.2	0.01	0.16	99	99
NAF –dna -1 –long 22	457.13	355	1.1	1920	2.2	0.02	0.17	99	99
JARVIS def.	274.86	18	0.1	71	0.6	0.30	0.30	339	1033
MBGC def.	589.13	2311	27.5	1989	4.2	20.03	0.42	492	135
FFC def.	116.03	6277	400.5	3275	38.9	0.10	0.04	1134	195
FFC –disable-long	102.16	6539	82.1	3269	10.2	0.09	0.04	891	196
uniprot_sprot (0.28 GB)									
pigz -1	2.75	1476	38.3	355	2.2	0.03	0.00	2602	138
igzip -1	2.94	2003	93.7	684	7.1	0.05	0.06	494	100
zstd -1	3.43	3505	108.0	1335	16.8	0.06	0.00	1016	142
zstd -1 –long=22	3.49	510	35.4	1335	25.1	0.06	0.01	244	144
pzstd -1	3.42	3115	117.0	3115	149.6	0.09	0.12	1187	434
NAF –text -1	4.14	346	5.3	904	15.3	0.02	0.09	99	99
NAF –text -1 –long 22	4.19	224	0.7	904	18.6	0.02	0.09	99	99
JARVIS def.	3.13	10	0.3	26	0.3	0.28	0.28	152	168
MBGC def.	4.17	11	0.0	66	2.3	2.02	1.15	262	227
FFC def.	3.89	2549	132.2	2336	196.9	0.17	0.07	894	304
FFC –disable-long	3.89	2549	111.8	2336	121.9	0.17	0.06	884	305
VDB (0.18 GB)									
pigz -1	3.06	1321	0.0	342	3.8	0.03	0.00	2566	137
igzip -1	3.34	1849	0.0	616	10.8	0.05	0.06	468	101
zstd -1	5.88	3082	122.1	1233	0.0	0.06	0.01	903	137
zstd -1 –long=22	7.03	544	42.4	1423	48.8	0.06	0.01	236	142
pzstd -1	5.78	3082	211.5	3082	193.1	0.09	0.11	1043	482
NAF –dna -1	14.01	420	3.5	1321	72.5	0.01	0.01	100	99
NAF –dna -1 –long 22	22.02	303	1.4	1423	45.1	0.01	0.01	99	99
JARVIS def.	36.71	13	0.2	12	0.0	0.32	0.32	163	105
MBGC def.	12.70	98	1.3	162	2.3	0.98	0.38	293	222
FFC def.	11.30	3082	122.1	3082	0.0	0.12	0.04	647	208
FFC –disable-long	10.11	3082	165.4	3082	193.1	0.11	0.05	589	210

The column “ratio” shows the ratio of the input to the output size. Median compression / decompression speeds are denoted as “cspeed” / “dspeed”, and are followed by their standard deviations over 13 runs. Compression and decompression peak memory usage is reported in columns “cmem” and “dmem”, respectively, while CPU usage during compression and decompression in columns “ccpu” and “dcpu”.

Table 6: Compression and decompression results. Max-performance scenario: cached input, output sent to the /dev/null virtual device.

	input size [GB]	pzstd -1, compression ratio	t1 [MB/s]	t4 [MB/s]	t8 [MB/s]	t12 [MB/s]	t28 [MB/s]	FFC def., compression ratio rel. pzstd	t1 [MB/s]	t4 [MB/s]	t8 [MB/s]	t12 [MB/s]	t28 [MB/s]
canFam6	2.36	2.94	248	918	1760	2510	3232	+30 %	545	2069	4718	5898	5754
felCat9	2.57	3.00	253	932	1799	2547	3341	+30 %	548	2075	4764	6125	5846
mouse	2.79	3.06	258	944	1833	2579	3356	+29 %	554	2079	4803	6055	5927
panTro3	3.37	3.35	280	1026	1928	2722	3552	+30 %	567	2149	4963	6249	6026
Danio rerio, zebrafish	1.7	3.00	256	939	1809	2538	3270	+17 %	518	1954	4360	5863	5668
CHM13v2.0_genomic	3.16	3.15	264	971	1868	2630	3431	+13 %	520	1985	4574	6189	6070
CHM13v2.0_rna	0.73	5.88	469	1653	3030	4041	4546	-30 %	622	2346	4849	6061	5595
hg19	3.2	3.19	267	979	1871	2623	3441	+30 %	571	2119	4923	6154	6038
GRCh38.p14_genomic	3.34	3.19	268	982	1876	2569	3443	+18 %	542	2012	4639	6301	6072
knownCanonical.exonNuc	0.34	5.02	396	1419	2433	3407	3785	+4 %	286	1065	2004	2620	3097
cere	0.49	3.35	274	988	1830	2600	3088	+30 %	633	2353	4941	5489	4941
T. aestivum	4.6	3.15	256	949	1835	2616	3436	+30 %	618	2314	5685	6873	6772
T. durum	3.39	3.21	261	965	1871	2646	3456	+28 %	515	1969	4576	6157	6271
D. melanogaster	8.73	4.93	437	1596	3086	4281	5229	+43 %	419	1667	3700	5390	6238
influenza	1.43	21.43	1143	3970	5497	5955	5955	+38 %	447	1722	3862	5497	5955
SARS-CoV-2	18.83	39.50	1895	5412	5655	5588	5689	+194 %	489	1904	4516	6539	7160
uniprot_sprot	0.28	3.42	438	1476	2804	3505	4005	+14 %	298	1168	2157	3115	3115
VDB	0.18	5.78	440	1423	2312	3082	3699	+96 %	462	1681	3082	3699	3082

	input size [GB]	pzstd -1, decompression ratio	t1 [MB/s]	t4 [MB/s]	t8 [MB/s]	t12 [MB/s]	t28 [MB/s]	FFC def., decompression ratio	t1 [MB/s]	t4 [MB/s]	t8 [MB/s]	t12 [MB/s]	t28 [MB/s]
canFam6	2.36	2.94	940	3188	6049	8425	9436	3.83	2184	11234	18147	21446	18147
felCat9	2.57	3.00	956	3341	6125	8870	9527	3.90	2218	11184	18374	21437	18374
mouse	2.79	3.06	961	3165	6055	8441	9948	3.95	2228	11606	19896	21427	18570
panTro3	3.37	3.35	1016	3341	6367	8653	10226	4.37	2250	12053	19851	22498	19851
Danio rerio, zebrafish	1.7	3.00	972	3208	6073	8502	9447	3.50	1977	9447	15458	18893	17004
CHM13v2.0_genomic	3.16	3.15	971	3254	6312	8767	9863	3.56	1998	10181	17535	21042	18566
CHM13v2.0_rna	0.73	5.88	1399	4546	7273	10390	10390	4.10	2909	12122	14546	18182	12122
hg19	3.2	3.19	985	3404	6274	8421	10000	4.16	2286	11852	20000	22857	20000
GRCh38.p14_genomic	3.34	3.19	988	3408	6301	8789	10120	3.75	2087	10773	17578	20873	19646
knownCanonical.exonNuc	0.34	5.02	1065	3407	5678	6813	6813	5.21	873	3407	5678	6813	5678
cere	0.49	3.35	932	3088	5489	7058	7058	4.37	2906	9881	16468	16468	12351
T. aestivum	4.6	3.15	921	3154	6059	8527	9797	4.09	2990	14854	24236	23024	20021
T. durum	3.39	3.21	951	3288	6157	8683	9960	4.12	1903	9960	17824	19920	16932
D. melanogaster	8.73	4.93	1460	4620	8911	12475	15320	7.04	1608	8397	13861	15877	14554
influenza	1.43	21.43	2598	7145	12992	17864	17864	29.49	2165	10208	17864	20416	15879
SARS-CoV-2	18.83	39.50	4176	10700	19414	25449	28108	116.03	2975	16235	28108	27694	22966
uniprot_sprot	0.28	3.42	1335	4005	7009	9345	7009	3.89	967	3505	5607	7009	5607
VDB	0.18	5.78	1321	3699	6164	9247	6164	11.30	2312	6164	9247	9247	6164

The top part of the table shows compression, while the bottom part decompression speeds. The columns "ratio" present the ratio of the input to the output size. In the top part of the table, instead of raw compression ratios for FFC, its values relative to pzstd ratios are given. The following columns show how the pzstd (mode -1) and FFC (default mode) speeds vary with the number of used threads from $\{1, 4, 8, 12, 28\}$ on the 14-core / 28-thread machine. FFC by default runs with 12 threads for compression and 4 threads for decompression. The default number of worker threads for pzstd equals to the available number of logical cores, both in its compression and decompression mode.
